# Hereditary and breastfeeding factors are positively associated with the aetiology of mammary gland hyperplasia: a case–control study

**DOI:** 10.1093/inthealth/ihaa028

**Published:** 2020-06-18

**Authors:** Hanlu Gao, Chao Yang, Jinqing Fan, Li Lan, Da Pang

**Affiliations:** Department of Preventive Health, The Affiliated Hospital of Medical School of Ningbo University, 247 Renmin Road, Ningbo, Zhejiang, P.R. China; Division of Chronic and Non-communicable Diseases, Harbin Center for Diseases Control and Prevention, 30 Weixing Road, Harbin, Heilongjiang, P.R. China; Department of Breast Surgery, Harbin Medical University Cancer Hospital, 150 Haping Road, Harbin, Heilongjiang, P.R. China; Division of Chronic and Non-communicable Diseases, Harbin Center for Diseases Control and Prevention, 30 Weixing Road, Harbin, Heilongjiang, P.R. China; Department of Dermatology, The Affiliated Hospital of Medical School of Ningbo University, 247 Renmin Road, Ningbo, Zhejiang, P.R. China; Division of Chronic and Non-communicable Diseases, Harbin Center for Diseases Control and Prevention, 30 Weixing Road, Harbin, Heilongjiang, P.R. China; Department of Breast Surgery, Harbin Medical University Cancer Hospital, 150 Haping Road, Harbin, Heilongjiang, P.R. China

**Keywords:** breastfeeding duration, family history of breast cancer, hyperplasia of mammary gland, interaction effect, reproductive factors

## Abstract

**Background:**

Hyperplasia of mammary gland (HMG) has become a common disorder in women. A family history of breast cancer and female reproductive factors may work together to increase the risk of HMG. However, this specific relationship has not been fully characterized.

**Methods:**

A total of 1881 newly diagnosed HMG cases and 1900 controls were recruited from 2012 to 2017. Demographic characteristics including female reproductive factors and a family history of breast cancer were collected. A multi-analytic strategy combining unconditional logistic regression, multifactor dimensionality reduction (MDR) and crossover approaches were applied to systematically identify the interaction effect of family history of breast cancer and reproductive factors on HMG susceptibility.

**Results:**

In MDR analysis, high-order interactions among higher-level education, shorter breastfeeding duration and family history of breast cancer were identified (odds ratio [OR] 7.07 [95% confidence interval {CI} 6.08 to 8.22]). Similarly, in crossover analysis, HMG risk increased significantly for those with higher-level education (OR 36.39 [95% CI 11.47 to 115.45]), shorter duration of breastfeeding (OR 27.70 [95% CI 3.73 to 205.70]) and a family history of breast cancer.

**Conclusion:**

Higher-level education, shorter breastfeeding duration and a family history of breast cancer may synergistically increase the risk of HMG.

## Introduction

Hyperplasia of mammary gland (HMG), a multifactorial complicated disease, accounts for >70% of all breast diseases that occur among middle-aged women and is highly associated with breast cancer.^1^ The prevalence of HMG is high in China, perhaps due to the quickening pace of life and increasing work-related pressure.^2^ Therefore, understanding the indicators of HMG in middle-aged women plays an important part in public health. Researchers have identified reproductive risk factors for HMG, such as late age at menopause, nulliparity and a lack of breastfeeding.^3^ Nevertheless, the aetiology of HMG remains largely unknown. A family history of breast cancer is an important indicator for women's risk of developing breast cancer.^4^ Recently there has been growing recognition that large sample sizes are needed in order to identify heredity variants that have effects modified by the environment as well.^5^ Heredity–environment interactions have the potential to illustrate the biologic causes of disease, distinguish individuals for whom risk factors are most related and develop precision medicine.^6^ However, few researchers have explored the interaction between a family history of breast cancer and HMG. Furthermore, existing studies include only a single statistical method to study the interaction between a family history of breast cancer and HMG, lacking the internal validation and decreased statistical power to identify underlying heredity–environment interactions.^7^

Using data collected in a large community-based case–control study, we assessed the correlation of HMG with a family history of breast cancer and reproductive factors in women self-reporting first- and second-degree relatives. We adopted multi-analytic strategy to scientifically examine the interactions between hereditary and female reproductive factors. Several statistical approaches, including traditional multiple logistic regression, multifactor dimensionality reduction (MDR) and crossover analysis were applied to explore the relationship between high-order hereditary and reproductive factors for HMG susceptibility.

## Methods

### Methods for recruiting samples

**Figure 1.**
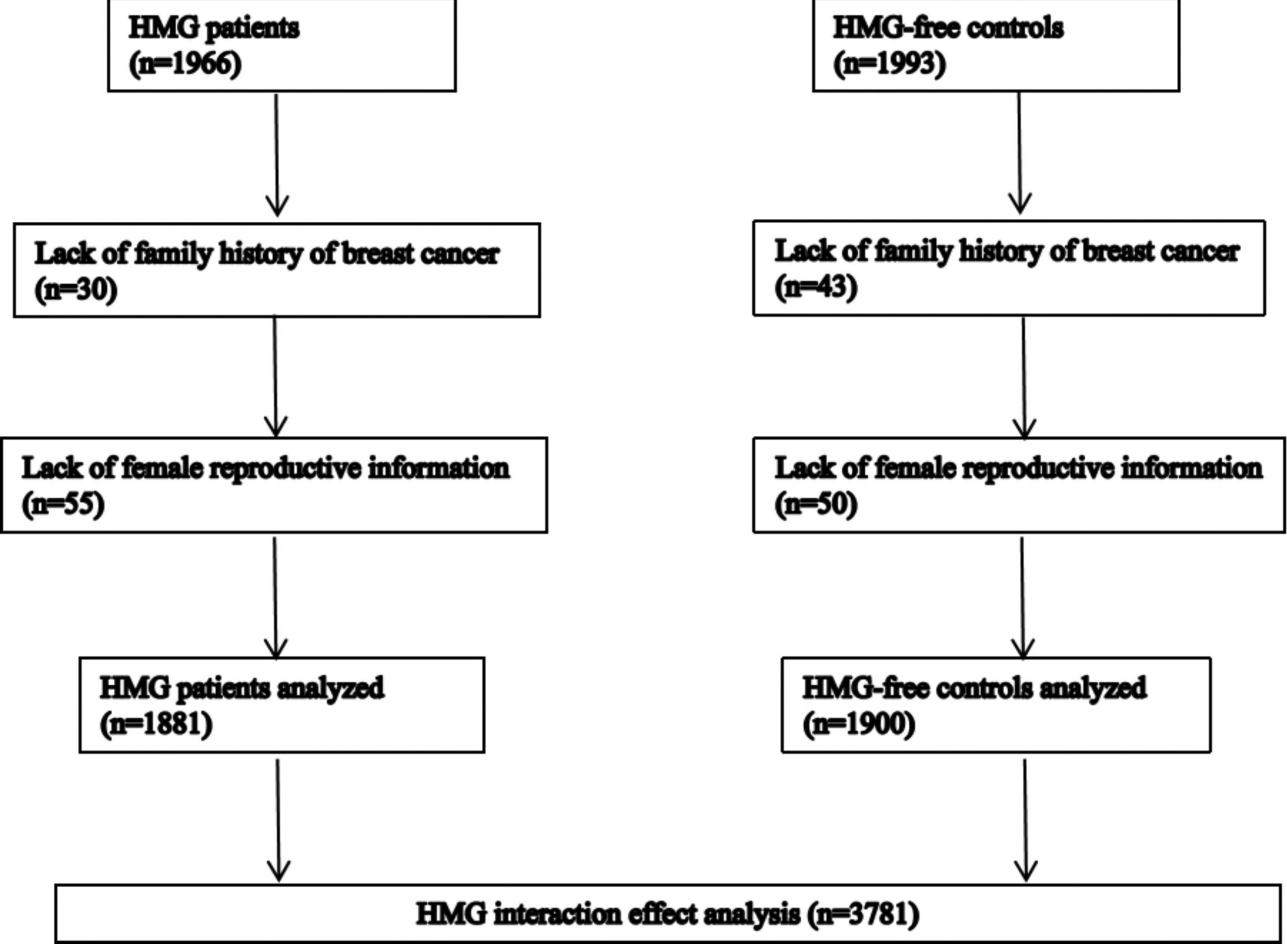
Flowchart of participant inclusion in the case–control study.

This study is based on the National Basic Public Health Service Project, which is provided free of charge for both urban and rural residents by the Chinese government. A total of 1966 patients who were newly diagnosed as HMG by colour Doppler ultrasonography from October 2012 to December 2017 were collected. Meanwhile, 1993 HMG-free controls were chosen from the community health service centre of Harbin. Inclusion criteria were female subjects newly diagnosed with HMG, age >35 y, living in Harbin for at least 6 months and who agreed to a colour Doppler ultrasound examination. Patients with mastitis, angiosarcoma, tumour of the mammary glands, breast cancer or other cancers were excluded. A total of 85 cases (4.4%) and 93 controls (4.7%) were excluded because of missing information and a total of 1881 cases and 1900 controls were enrolled (Fig. 1). The clinical results were reviewed by two general practitioners to ensure the diagnosis. All participants provided informed consent and the study was approved by the Ethical Committee of Harbin Center for Disease Control and Prevention.

### Data collection

Basic demographic information (including age, ethnicity, education level, marriage and occupation) and female reproductive factors (including menopausal status, age at menopause, parturition and age at first delivery, age at menarche, breastfeeding and its duration and family history of breast cancer) were obtained using a structured questionnaire administered by trained interviewers face to face. In this research, history of breast cancer was defined as breast malignancy in a first- or second-degree relative (mother, sister, grandmother or aunt). Regular menstruation was considered as a menstrual time of 2–7 d and a menstrual cycle of 24–35 d. Menopause referred to the specific period from the appearance of endocrine, biological and clinical characteristics related to menopause to the postmenopausal period. Fibrous (cystic) and single-type HMG was included in our study. According to the fifth edition of the American College of Radiology (ACR) Breast Imaging Reporting and Data System (BI-RADS) guidelines, cases classified as stages II–V were included in our study (BI-RADS 2: normal; BI-RADS 3: benign lesions; BI-RADS 4: suspicion for malignancy; BI-RADS 5: highly suggestive of malignancy). The lesion site (left breast, right or bilateral) was taken used as the BI-RADS grade.

### Statistical analyses

The odds ratios (ORs) and corresponding 95% confidence intervals (CIs) were summarized to estimate the associations between reproductive factors and HMG risk by univariate and manual stepwise multivariate logistic regression. All references values for exposure were the lower level of the variables. Interactions between a family history of breast cancer and female reproductive factors were evaluated by MDR. The MDR approach includes a cross-validation procedure that minimizes the possibility of false-positive results by dividing the data into a testing set and a training set. Cross-validation consistency (CVC) provided a summary for the number of cross-validation intervals for discovering a particular model. Higher numbers mean more stable results. The joint effects between female reproductive factors and a family history of breast cancer on the risk of HMG were analysed by the crossover method. Additive interactions were calculated by the relative excess risk of interaction (RERI), attributable proportions of interaction (API) and the synergy index (SI) as described by Andersson et al.^8^ The potential confounding variables were controlled in the process of analysing the interactions. P-values <0.05 were considered statistically significant and all p-values were two-tailed. All statistical analyses were performed with SPSS Statistics (version 21.0; IBM, Armonk, NY, USA), SAS (version 9.2; SAS Institute, Cary, NC, USA) and MDR software (Unix, version 2.0; The Open Group, San Francisco, CA, USA).

## Results

### Basic characteristics of HMG patients and controls

The female reproductive factors and ORs for HMG are presented in Table 1. Of the 1881 cases, 1627 were Han ethnicity and 1203 of the 1900 controls were of Han ethnicity. The difference in mean ages between cases (51.27±6.62) and controls (51.21±6.62) was not significant (t = 0.27, p = 0.89). Among the study population, distributions of body mass index (BMI), education, marriage and occupation type were significantly different between cases and controls (p < 0.05). Cases tended to be more educated, married, older age at first delivery and had a shorter breastfeeding duration, less breastfeeding and regular menstruation and parturition than controls. Age at menopause was also significantly different between cases and controls in the 50- to 54-y age group. According to BI-RADS, 1646 cases were classified as stage II, 192 as stage III, 38 as stage IV and 5 as stage V HMG. BMI and occupation were treated as potential confounders and adjusted in crossover, multiplicative interaction and additive interaction analyses.

**Table 1.** Basic characteristics of HMG patients and controls

Effect factors	Patients, n (%)	Controls, n (%)	OR (95% CI)	p-Value
Age (years)				
35–44	324 (17.2)	331 (17.4)	1	
45–54	1002 (53.3)	1015 (53.4)	1.01 (0.85 to 1.20)	0.93
55–64	488 (25.9)	487 (25.6)	1.02 (0.84 to 1.25)	0.82
≥65	67 (3.6)	67 (3.5)	1.02 (0.70 to 1.48)	0.91
BMI (kg/m^2^)				
<18.5	31 (1.7)	18 (1.0)	1	
18.5–23.9	1073 (57.4)	1120 (59.3)	0.56 (0.31 to 1.00)	0.05
24–27.9	646 (34.5)	658 (34.8)	0.57 (0.32 to 1.03)	0.06
≥28	120 (6.4)	93 (4.9)	0.75 (0.40 to 1.42)	0.38
Nationality				
Han people	1627 (97.8)	1203 (97.6)	1	
Other	37 (2.2)	29 (2.4)	0.94 (0.58 to 1.54)	0.82
Education level				
High school or below	1452 (77.2)	1745 (91.8)	1	
College or above	429 (22.8)	155 (8.2)	3.33 (2.73 to 4.05)	<0.01
Marriage				
Unmarried	19 (1.0)	5 (0.30)	1	
Married	1862 (99.0)	1895 (99.7)	0.26 (0.10 to 0.69)	<0.01
Occupation				
White collar	892 (47.4)	372 (19.6)	1	
Blue collar	989 (52.6)	1528 (80.4)	0.27 (0.23 to 0.31)	<0.01
Regular menstruation				
No	633 (33.7)	231 (12.2)	1	
Yes	1248 (66.3)	1669 (87.8)	0.27 (0.23 to 0.32)	<0.01
Age at menarche (years)				
0–11	49 (2.6)	37 (1.9)	1	
≥12	1832 (97.4)	1863 (98.1)	0.74 (0.48 to 1.14)	0.18
Breastfeeding				
No	346 (19.1)	104 (5.5)	1	
Yes	1461 (80.9)	1792 (94.5)	0.25 (0.2 to 0.31)	<0.01
Breastfeeding duration (months)				
0–6	187 (12.8)	69 (3.9)	1	
≥7	1274 (87.2)	1723 (96.1)	0.27 (0.21 to 0.36)	<0.01
Menopause				
Premenopausal	1003 (53.3)	973 (51.2)	1	
Postmenopausal	878 (46.7)	927 (48.8)	0.92 (0.81 to 1.04)	0.19
Age at menopause (years)				
35–49	317 (36.1)	272 (29.3)	1	
50–54	520 (59.2)	630 (68.0)	0.71 (0.58 to 0.86)	<0.01
≥55	41 (4.7)	25 (2.7)	1.41 (0.83 to 2.37)	0.20
Parturition				
No	109 (5.8)	2 (0.10)	1	
Yes	1769 (94.2)	1897 (99.9)	0.02 (0.01 to 0.07)	<0.01
Age at first delivery (years)				
20–24	439 (24.8)	619 (32.6)	1	
≥25	1330 (75.2)	1278 (67.4)	1.47 (1.27 to 1.70)	<0.01
Family history of breast cancer				
No	1414 (75.2)	1885 (99.2)	1	
Yes	467 (24.8)	15 (0.8)	41.50 (24.71 to 69.92)	<0.01

ORs (95% CIs) and p-values were calculated by univariate logistic regression.

### Associations between female reproductive factors and risk of HMG

Married women, manual workers, regular menstruation, breastfeeding history, longer breastfeeding duration, early age at menopause and early parturition had 0.26, 0.27, 0.27, 0.25, 0.27, 0.71 and 0.02-fold reductions in the risk of HMG when compared with controls. A significant increase in HMG risk was associated with later age at first delivery and family history of breast cancer (OR 1.47 [95% CI 1.27 to 1.70] and OR 41.50 [95% CI 24.71 to 69.92], respectively; Table 1).

After multifactor unconditional logistic regression modelling, we found that education level, BMI, age at first delivery and family history of breast cancer were statistically positively associated with HMG (OR 1.62 [95% CI 1.27 to 2.07], OR 1.15 [95% CI 1.01 to 1.30], OR 1.33 [95% CI 1.11 to 1.59], OR 37.87 [95% CI 22.33 to 64.20], respectively), whereas occupation type and breastfeeding duration were statistically negatively associated with HMG (OR 0.30 [95% CI 0.25 to 0.36, OR 0.34 [95% CI 0.25 to 0.46], respectively; Table 2).

**Table 2.** Multiple logistic regression analysis of influencing factors of HMG

Factors	OR (95% CI)	p-Value
Education level	1.62 (1.27 to 2.07)	<0.01
Occupation type	0.30 (0.25 to 0.36)	<0.01
BMI	1.15 (1.01 to 1.30)	0.04
Breastfeeding duration	0.34 (0.25 to 0.46)	<0.01
Age at first delivery	1.33 (1.11 to 1.59)	<0.01
Family history of breast cancer	37.87 (22.33 to 64.20)	<0.01

### Female reproduction and family history of breast cancer interactions and the risk of HMG

Table 3 displays the CVC from the one- to four-factor models for each situation. The three-factors model including education level, breastfeeding duration and family history of breast cancer had a maximum testing accuracy of 71.1% and a maximum CVC of 100%. Therefore this model was regarded as the best among all the interaction models calculated by MDR. As Table 4 shows, compared with the ‘low-risk’ combinations, participants classified as ‘high-risk’ combinations significantly increase HMG risk by 7.07-fold (95% CI 6.08 to 8.22).

**Table 3.** Analysis of MDR results

Model	Training balance accuracy	Testing balance accuracy	CVC
Family history of breast cancer	0.62	0.62	10/10
Breastfeeding duration/family history of breast cancer	0.69	0.69	10/10
Education level/breastfeeding duration/ family history of breast cancer	0.71	0.71	10/10
Education level/age at first delivery/ breastfeeding duration/family history of breast cancer	0.71	0.71	10/10

**Table 4.** Details of the optimal model based on MDR

Indicators	Training dataset statistics	Testing dataset statistics	Whole dataset statistics
Balanced accuracy	0.71	0.71	0.71
Accuracy	0.71	0.71	0.71
Sensitivity	0.59	0.59	0.59
Specificity	0.83	0.83	0.83
OR (95% CI)	7.07 (6.03 to 8.29)	7.07 (4.39 to 11.39)	7.07 (6.08 to 8.22)
χ^2^	643.06	71.45	714.51
p-Value	<0.01	<0.01	<0.01
Precision	0.78	0.78	0.78
κ	0.42	0.42	0.42
F measure	0.67	0.67	0.67
Cross-validation consistency	10/10		

### Multiplicative interactions between female reproductive factors and family history of breast cancer on the risk of HMG

We did not find statistically significant multiplicative interactions between education level (OR 0.43 [95% CI 0.11 to 1.59], p = 0.20), breastfeeding duration (OR 1.42 [95% CI 0.18 to 11.38], p = 0.74), the interaction effect of education level and breastfeeding duration (OR 0.67 [95% CI 0.35 to 1.30], p = 0.24) and family history of breast cancer on HMG (Table 5).

**Table 5.** The multiplier interaction between family history of breast cancer and environmental factors

	Family history of breast cancer	
Factors	OR (95% CI)	p-Value
Education level	0.43 (0.11 to 1.59)	0.20
Breastfeeding duration	1.42 (0.18 to 11.38)	0.74
Education level and breastfeeding duration	0.67 (0.35 to 1.30)	0.24

ORs adjusted for BMI and occupation type.

### The combination effect between female reproduction and family history of breast cancer on the risk of HMG

Significant individual and joint effects between education level, breastfeeding duration and family history of breast cancer were detected (Table 6). The coexistence of a family history of breast cancer and higher-level education increased the risk of HMG to 36.39 (95% CI 11.47 to 115.45), higher than the individual risks associated with higher-level education alone (OR 1.96 [95% CI 1.57 to 2.46]) but lower than the individual risk associated with a family history of breast cancer (OR 46.52 [95% CI 25.97 to 83.32]). The combination of a family history of breast cancer and breastfeeding duration were associated with a markedly increased risk for HMG (OR 12.74 [95% CI 6.85 to 23.71]).

**Table 6.** Crossover analysis in assessing the association between family history of breast cancer and environmental factors for HMG

Factors	Patients, n (%)	Controls, n (%)	Total, n (%)	Prevalence of breast hyperplasia (%)	OR (95% CI)
Family history of breast cancer/education level					
No/low	1103 (58.6)	1733 (91.2)	2836 (75.0)	38.89	1
No/high	311 (16.5)	152 (8)	463 (12.2)	67.17	1.96 (1.57 to 2.46)
Yes/low	349 (18.6)	12 (0.6)	361 (9.5)	96.68	46.52 (25.97 to 83.32)
Yes/high	118 (6.3)	3 (0.2)	121 (3.2)	97.52	36.39 (11.47 to 115.45)
Family history of breast cancer/breastfeeding duration					
No/low	130 (8.9)	68 (3.7)	198 (6.09)	65.66	1
No/high	966 (66.12)	1709 (95.37)	2675 (82.23)	36.11	0.33 (0.24 to 0.45)
Yes/low	57 (3.9)	1 (0.06)	58 (1.78)	98.28	27.70 (3.73 to 205.70)
Yes/high	308 (21.08)	14 (0.78)	322 (9.90)	95.65	12.74 (6.85 to 23.71)

ORs adjusted for BMI and occupation type.

### The additive effect between female reproduction and family history of breast cancer on the risk of HMG

Because the combinations of a family history of breast cancer and breastfeeding duration and a family history of breast cancer and education level were found in joint effects, their additive effects were analysed. The ORs and 95% CIs of the relative excess risk of interaction (RERI), attributable proportions of interaction (API) and synergy index (SI) are indicators for additive interactions. There were no statistically significant additive interactions between education level, breastfeeding duration and family history of breast cancer on the risk of HMG (Table 7).

**Table 7.** Additive interaction between family history of breast cancer and environmental factors

Factors	RERI, OR (95% CI)	API, OR (95% CI)	SI, OR (95% CI)
Family history of breast cancer/education level	−11.09 (−60.92 to 38.74)	−0.31 (−1.98 to 1.37)	0.76 (0.20 to 2.85)
Family history of breast cancer/breastfeeding duration	−14.29 (−69.79 to 41.23)	−1.12 (−5.58 to 3.34)	0.45 (0.05 to 4.03)

ORs adjusted for BMI and occupation type.

## Discussion

HMG, characterized by breast pain and lumps, is a common disease in women. Endocrine disorders,^9^ mental factors^10^ and genetic factors^11^ have been confirmed to impact HMG. Treatments for HMG include hormone replacement drugs,^12^ traditional Chinese medicine^13^ and lifestyle interventions.^14^ However, the pathogenesis of HMG is still unclear.

In this case–control study of HMG, evidence was found that the risk of HMG is influenced not only by a family history of breast cancer but also by breastfeeding duration and education level. Possible interactions between hereditary and reproductive factors of HMG were noted. Various algorithms were used to explore the interactions between a family history of breast cancer and female reproductive factors. First, MDR was used to analyse the interactions of six environmental factors that were statistically significant in multiple logistic regression. High-dimensional interactions, including education level, breastfeeding duration and family history of breast cancer, were detected. Second, we adopted a crossover analysis method and found a strong synergistic effect between a family history of breast cancer and higher education level after adjusting for BMI and occupation. Therefore more attention should be paid to enhancing awareness and health education among HMG women with higher education levels and a family history of breast cancer.^15^ Additionally, an antagonistic effect between a family history of breast cancer and breastfeeding duration was also observed, which was consistent with the published literature.^16^ Based on these results, women with a family history of breast cancer may reduce their risk of HMG through adjustments in reproductive choices.^17^ Third, since the additive model might be better to explain the biologic interaction, we also estimated the RERI, API and SI by additive models, but we did not find a statistical difference. Sample sizes may have led to a reduction in statistical power.^18^ Although we did not find an effect of education level or breastfeeding duration combined with a family history of breast cancer, several lines of evidence suggest that our findings are biologically plausible. Our research also found that HMG individuals with a family history of breast cancer had a greater chance of developing neoplasia.^19^ The activation of Akt-1, which peaks in lactation, regulates survival of epithelial cells. A shorter breastfeeding duration decreased Akt-1 significantly, which my contribute to HMG.^20^ A higher education level is often accompanied by high stress, which is thought to be connected with an increased risk of breast disease.^21^ Normal growth of the mammary gland involves endocrine signals from the hypothalamic–pituitary–gonadal axis.^22^ Stress has been shown to disrupt the function of the endocrine system and increase susceptibility to HMG.^23^ Additionally, an increasing level of inflammatory burden and hypothalamic–pituitary–adrenocortical axis dysregulation subsequent to stress may also cause HMG.^24^ These observations indicate that heredity–environment interactions might be especially important for HMG.^25^ Therefore HMG prevention strategies should be individualized according to an individual's exposure to risk factor profiles.

Heredity–environment interactions are consistently distinguished by both non-parametric and parametric statistical models. Logistic regression has the advantage of analysing for the main effect. When high-order interactions involving multidimensional elements are taken into account, they may be limited in dealing with simultaneous factors.^26^ MDR can identify putative high-order interactions, but is limited in analysing main effects in many diseases.^27^ Crossover analysis can evaluate the independent and joint roles of genetics and exposure on disease hazard.^28^ However, it can only analyse the interactions between binary variables.^29^ Recent studies have shown that multiple complementary analytical strategies, including logistic regression and MDR, could improve statistical power to identify underlying heredity–environment interactions.^30,31^

Results from MDR and crossover analysis consistently show that a family history of breast cancer is the most significant single risk for HMG and HMG risk is substantially associated with education level and breastfeeding duration interactions. In this research, the MDR and crossover analysis validated each other and emphasized the repeatability of our results.

Nevertheless, this study still has some limitations. The association between a family history of breast cancer and female reproductive factors was analysed. Further studies are imperative to understand whether the interactions are related to other factors such as dietary habits, lifestyle and hormone replacement therapy. Furthermore, the results obtained in this research could be affected by recall bias, which frequently appears in case–control studies, thus replication in other independent samples of observed interactions is needed to verify our results. Moreover, the number of cases in the strata was relatively small. Therefore these variables may not be adequately powered to assess interactions. Expanding the sample size or finding other more applicable statistical analysis methods to analyse interactions is needed in future studies. Lastly, all patients with HMG did not have a tissue biopsy, so the related mechanism of patients with different types of HMG is needed in further research.

## Conclusions

High-order interactions of higher-level education, shorter breastfeeding duration and a family history of breast cancer might synergistically increased HMG risk.
